# High prevalence of mental disorder symptoms among medical and other health specialties residents during the COVID-19 pandemic

**DOI:** 10.1186/s12909-023-04202-7

**Published:** 2023-05-22

**Authors:** Nayane Miranda Silva, Rebeca da Nobrega Lucena Pinho, Thais Ferreira Costa, Adriana Ferreira Barros Areal, André De Mattos Salles, Andrea Pedrosa Ribeiro Alves Oliveira, Carlos Henrique Reis Esselin Rassi, Caroline Elizabeth Brero Valero, Ciro Martins Gomes, Dayde Lane Mendonça da Silva, Fernando Araujo Rodrigues de Oliveira, Isadora Jochims, Ivan Henrique Ranulfo Vaz Filho, Juliana De Brito Seixas Neves, Lucas Alves de Brito Oliveira, Maria Luisa Nogueira Dantas, Marta Alves Rosal, Mayra Veloso Ayrimoraes Soares, Patrícia Shu Kurizky, Viviane Cristina Uliana Peterle, Yasmin Furtado Faro, Ana Paula Monteiro Gomides, Licia Maria Henrique da Mota, Cleandro Pires de Albuquerque, Cezar Kozak Simaan, Veronica Moreira Amado

**Affiliations:** 1 Graduate Program in Medical Sciences, Faculty of Medicine, University of Brasília-UnB - Darcy Ribeiro University Campus - Asa Norte, Brasília, 910-900 DF Brazil; 2 University Hospital of Brasília (HUB)-UnB, North Large Area Sector 605 - Asa Norte, Postal, Code, Brasília, 70840-901 DF Brazil; 3 State Health Department of the Federal District (SES DF), Central Administration: North Radio and TV Sector (SRTVN), 701 North, Postal Code 70.719-040, Brasília, Brazil; 4 University of Brasília (UnB) - Darcy Ribeiro University Campus - Asa Norte, 70.910-900, Brasília, DF Brazil; 5 Sírio-Libanês Hospital, SGAS 614/615, Postal Code, Asa Sul, Brasília, 70200-730 Brazil; 6 Empresa Brasileira de Serviços Hospitalares (EBSERH), SCS Quadra 6 Block A, Postal Code, Asa Sul, Brasília, 70308-200 Brazil; 7 Center for Tropical Medicine, University of Brasília-UnB - Darcy Ribeiro University Campus - Asa Norte, Brasília, 910-900 DF Brazil; 8 Faculty of Medicine, University of Brasília-UnB - Darcy Ribeiro University Campus - Asa Norte, Brasília, 910-900 DF Brazil; 9 Federal University of Piauí (UFPI), Minister Petrônio Portella University Campus, Postal Code, Ininga, 64049-550 Teresina Brazil; 10 School of Health Sciences (ESCS), SMHN Conjunto A Block 01 Fepecs Building, Postal Code, Asa Norte, Brasília, 70710-907 Brazil; 11 Brasília University Centre (Uniceub), 707/907 North, University Campus, Postal Code, Asa Norte, Brasília, 70790- 075 Brazil

**Keywords:** Mental health, Medical residency, Multiprofessional residency, COVID-19, Stress, Anxiety, Depression, Health care professional, Medical student

## Abstract

**Background:**

The COVID-19 pandemic put healthcare professionals, including residents (postgraduate trainees of health professions), under intense physical and psychological stress, hence at risk for mental disorders. We evaluated the prevalence of mental disorders among healthcare residents during the pandemic.

**Methods:**

From July to September 2020, residents in medicine and other healthcare specialties in Brazil were recruited. The participants completed electronic forms with validated questionnaires (DASS-21, PHQ-9, BRCS) to screen for depression, anxiety, and stress, and to evaluate resilience. Data on potential predisposing factors for mental disorders were also collected. Descriptive statistics, chi-squared, students t, correlation and logistic regression models were applied. The study received ethical approval, and all participants provided informed consent.

**Results:**

We included 1313 participants (51.3% medical; 48.7% nonmedical) from 135 Brazilian hospitals; mean (SD) age: 27.8 (4.4) years; 78.2% females; 59.3% white race. Of all participants, 51.3%, 53.4% and 52.6% presented symptoms consistent with depression, anxiety, and stress, respectively; 61.9% showed low resilience. Nonmedical residents exhibited higher anxiety compared to medical residents (DASS-21 anxiety score, mean difference: 2.26; 95% CI: 1.15–3.37; p < 0.001). In multivariate analyses, having any pre-existent, nonpsychiatric chronic disease was associated with higher prevalence of symptoms indicative of depression (odds ratio, OR: 2.05; 95% CI: 1.47–2.85, on DASS-21 | OR: 2.26; 95% CI: 1.59–3.20, on PHQ-9), anxiety (OR: 2.07; 95% CI: 1.51–2.83, on DASS-21), and stress (OR: 1.53; 95% CI: 1.12–2.09, on DASS-21); other predisposing factors were identified; by contrast, high resilience (BRCS score) was protective against symptoms of depression (OR 0.82; 95% CI: 0.79–0.85, on DASS-21 | OR 0.85; 95% CI: 0.82–0.88, on PHQ-9), anxiety (OR 0.90; 95% CI: 0.87–0.93, on DASS-21), and stress (OR 0.88; 95% CI: 0.85–0.91, on DASS-21); p < 0.05 for all outcomes.

**Conclusions:**

We found a high prevalence of mental disorder symptoms among healthcare residents during COVID-19 pandemic in Brazil. Nonmedical residents exhibited higher levels of anxiety than medical ones. Some predisposing factors for depression, anxiety and stress among residents were identified.

**Supplementary Information:**

The online version contains supplementary material available at 10.1186/s12909-023-04202-7.

## Background

The SARS-CoV-2 or coronavirus disease (COVID-19) epidemic was first identified in Wuhan, China, at the end of 2019 and then spread worldwide in a rapid and disordered manner [1, 2]. On March 11, 2020, the WHO characterized the spread of the disease as a pandemic, given its geographic extent [2]. In Brazil, the first case was recorded on February 25, 2020 [3, 4]. In January 2022, the cumulative recorded deaths exceeded 600,000 [5]. Brazil faces immense challenges regarding COVID-19 because of the country’s vast area, the high population density in some cities, the wide variety of air, land and sea routes connected to the world and a health system with limited resources [4, 6].

Given this scenario, health professionals, including residents in medicine and other health specialties who were required to cope with the disease [7], began to experience intense physical and psychological pressure daily [8]. Factors such as work overload, staff shortages, the prolonged use of personal protective equipment, sleep deprivation, limited knowledge about the disease, and the lack of specific drugs for treatment predispose this population to the development of mental disorders and psychological distress [9].

Recent studies have shown a high prevalence of mental disorders among health professionals attributed to intense emotional demands and adverse working conditions [9]. However, studies are scarce in the subgroup of professionals in training who compose residency programmes in medicine and other health care areas [10].

Residency programs are characterized by in-service training under supervision, with purpose of developing professional skills and competencies [11]. In Brazil, it constitutes a postgraduate teaching modality, *latu sensu*, which main characteristic is in health service training. In this process, configured by the worker-apprentice duality, the medi*c*al or multidisciplinary resident faces a constant internal tension that can both help in their professional improvement and act as a triggering factor for mental disorders [11–13].

The literature points to the recognition of the importance of these programs as a way of preparing for work, by allowing the confrontation of real situations permeated by the exchange of experiences with preceptors and other service professionals. However, it also alerts to the need for systematic monitoring of the residents’ quality of life in terms of physical, environmental, psychological and relational aspects, due to the extensive (60 h per week) and intense workload [14].

In addition to the challenges inherent to their professions, these professionals are pressured to acquire new knowledge and skills quickly and efficiently and are exposed to situations that require important decision making, which until now has not been their responsibility [9, 15]. In addition, these individuals commonly need to supplement their income, received as part of their scholarship, thus extending their workday in unsupervised services [15].

The mental health of resident physicians is a topic that had already preoccupied medical educators around the world even before the emergence of the pandemic. In 2014, the prevalence rates of mental disorders among Brazilian residents were 41.3% and 21.6% for depression and anxiety, respectively [16].

There are few studies on mental disorders in residents in medicine and other specialties during the COVID-19 pandemic, and to date, no study that simultaneously addresses medical residency and other health specialties has been conducted in Brazil. Understanding the impact of this pandemic on these residents’ mental health is essential to addressing the issue and planning health actions.

The aim of this study is to evaluate the prevalence of symptoms indicative of mental disorders such as depression, anxiety and stress among postgraduate students in medicine and other health discipline residencies during the COVID-19 pandemic in Brazil and identify possible associated predisposing factors.

## Methods

This study adopted a cross-sectional design that included postgraduate students from medical and other health residency programmes in Brazil. The students were over 18 years of age, assigned to activities involving the direct provision of care to patients (with or without COVID-19) and agreed to participate by signing an informed consent form. In Brazil, there are residency programmes for not only physicians, dentists and pharmacists but also several other categories of health professionals, and all categories were eligible for this study in such a way that facilitated evaluation of the professional residencies in health as a whole. The recruitment period extended from July 29 to September 5, 2020. For convenience with regard to participant recruitment, the study focused primarily on residents of federal university hospitals. An invitation to complete the study questionnaires were sent by email and social networks to the 7,215 residents of university hospitals affiliated with the Brazilian Hospital Services Company (Empresa Brasileira de Serviços Hospitalares – EBSERH). Dissemination also occurred through banners and announcements on the intranet of the university hospitals. However, the study was not restricted to residents affiliated with the EBSERH. Given the wide potential of dissemination via electronic social networks, residents of any Brazilian hospital were eligible and allowed to participate.

The participants completed an electronic form via the Microsoft Forms platform, through which epidemiological and clinical data were collected, including evaluations of psychological and affective aspects, following a predefined protocol [17] and using the following instruments, whose cutoff points between normal and abnormal are based on validation studies for the Portuguese language, according to the references cited below:

1. Depression Anxiety Stress Scale-21 (DASS-21). This instrument, translated into and validated for Portuguese [18], is composed of three subscales that measure three domains of symptoms, i.e., depression, anxiety and stress, with cutoff scores of > 9, > 7 and > 14, respectively, for the “abnormal” category.
2. Patient Health Questionnaire 9-item depression module (PHQ-9). This instrument has been translated into Portuguese and validated for Brazil [19]. It is composed of nine questions that evaluate the frequency of certain symptoms, i.e., 0 (not at all), 1 (several days), 2 (more than half the days) and 3 (nearly every day), with a cutoff point > 9 for abnormal values.
3. Brief Resilient Coping Scale (BRCS). The BRCS is a unidimensional instrument consisting of four items that assess the ability to adaptively cope with stress. A score < 13 is classified as “low resilient coping” [20].
4. A visual numerical scale was employed to evaluate each resident’s perception of their degree of autonomy at work. The response options ranged from 0 to 10, with zero corresponding to “I have no autonomy” and ten corresponding to “I have full autonomy”. A value ≤ 4 indicated low autonomy at work [21].
5. A visual numerical scale was created to assess the resident’s perception of the pedagogical structure of the medical or multiprofessional residency programme. The response options ranged from 1 to 10, with 1 corresponding to “totally inadequate” and 10 corresponding to “totally adequate”, with the cutoff score set to ≤ 5.
6. A Likert scale was created to assess each individual’s perception of the availability of personal protective equipment (PPE). The following question was posed: “In your professional practice, in patient care, what fraction of the time do you have enough and adequate PPE at your disposal?”. The response options were (1) “at no time”; (2) “less than half the time”; (3) “half the time”; (4) “more than half the time” and (5) “all the time”. The cutoff score was set to ≤ 3.
7. One question inquired whether students engaged in professional work outside the medical or nonmedical residency training programme: “yes” or “no”.
8. One question asked whether students provided direct care provided to patients with COVID-19: “yes” or “no”.

### Sample size calculation

For the purpose of sample size calculation, the prevalence of depressive disorder among health care professional residents (as assessed by moderate to high scores on the PHQ-9) was taken as the primary outcome. The expected prevalence of depressive disorder was set to 17%, similar to that reported among medical students, residents and fellows in a study conducted at a university hospital in New York during the COVID-19 pandemic [22]. Thus, assuming a large (“infinite”) target population, aiming for a 95% confidence interval with an allowable error (margin) of ± 0,03, based on a one-proportion Z test, the minimal sample size was estimated at N = 603 participants. The calculations were conducted through EPITOOLS (https://epitools.ausvet.com.au/oneproportion)

### Statistical analysis

The general characterization of the sample was performed using descriptive techniques, reporting absolute and relative frequencies for categorical variables and measures of central tendency and dispersion for continuous numerical variables. In bivariate analyses, associations between dichotomous categorical variables were determined using the chi-square test, with estimates of effect size by the odds ratio. Differences between medical and nonmedical residents regarding continuous variables were determined using Student’s t test, with Levene’s test for unequal variances and Welch’s correction applied when appropriate (unequal variances identified). Tests for the normality of the data distribution for continuous variables were applied. However, considering the sample size, by the central limit theorem, parametric approaches were applied in all cases in our analyses. Correlations between the scores for depression, anxiety and stress on the PHQ-9 and DASS-21 instruments and their subscales were determined using the Pearson r coefficient. Binomial logistic regression models were fitted to classify residents regarding the status of depression, anxiety and stress using the DASS-21 and PHQ-9 instruments, with independent evaluations of the contribution of several candidate predictor variables. The candidate predictor variables identified as significant (p < 0.05) in the bivariate analysis were included in multivariate analysis.

### Ethical aspects

All participants signed an informed consent form, and the study was approved by the Research Ethics Committee of the School of Medicine of the University of Brasília (CAAE no. 33493920.0.0000.5558) through the Research Ethics Committee/National Research Ethics Commission system (CEP/CONEP, acronym in Portuguese).

## Results

In total, 1,313 residents in medicine and other health specialties participated in the study. The general characteristics of the studied sample are shown in Table 1.

**Table 1:** General characteristics of the sample

CHARACTERISTIC			Total n = 1313
**GENDER (1310 responses)**			
Female			1025 (78.2%)
Male			285 (21.8%)
**RACE (1313 responses)**			
White			778 (59.3%)
Nonwhite			535 (40.7%)
**TYPE OF INSTITUTION (1313 responses)**			
Public			1270 (96.7%)
Private or Philanthropic			43 (3.3%)
**UNIVERSITY HOSPITAL (1313 responses)**			
Yes			1177 (89.6%)
No			136 (10.4%)
**TYPE OF RESIDENCY PROGRAM (1313 responses)**			
Medical			674 (51.3%)
Nonmedical			639 (48.7%)
**PROFESSIONAL CATEGORY OF THE PARTICIPANT (1272 responses)**			
Doctor			674 (53.0%)
Nurse			115 (9.0%)
Pharmacist			91 (7.2%)
Nutritionist			82 (6.4%)
Psychologist			82 (6.4%)
Physical therapist			63 (5.0%)
Social worker			51 (4.0%)
Dentist			37 (2.9%)
Occupational therapist			22 (1.7%)
Other			55 (4.3%)
**PROVIDING DIRECT CARE TO PATIENTS WITH COVID-19 (1313 responses)**			
Yes			790 (60.2%)
No			523 (39.8%)
**PRESENCE OF DISEASES (1305 responses)**			
Yes			234 (17.9%)
No			1071 (82.1%)
**INCREASED RISK FOR SERIOUS FORMS OF COVID-19 (1305 responses)**			
Yes			218 (16.7%)
No			1087 (83.3%)
**PERCEPTION OF PPE AVAILABILITY (1313 responses)**			
Poor			281 (21.4%)
Moderate to good			1032 (78.6%)
**PERCEPTION OF THE PEDAGOGICAL ORGANIZATION OF THE RESIDENCY PROGRAM**			
**(1313 responses)** Poor			558 (42.5%)
Moderate to good			755 (57.5%)
**AUTONOMY AT WORK (1313 responses)**			
Low autonomy			224 (17.1%)
Moderate to high autonomy			1089 (82.9%)
**WEEKLY WORKLOAD (1313 responses)**			
≤ 60 h			541 (41.2%)
≥ 60 h			772 (58.8%)
**WORK OUTSIDE THE RESIDENCY PROGRAMME (1313 responses)**			
Yes			424 (32.3%)
No			889 (67.7%)

*PPE: personal protective equipment

Most residents were female (78.2%), and the mean (SD) age was 27.8 (4.4) years. Regarding ethnicity, 59.3% reported being white, 33% mixed race, 6.2% black and 1.6% “other”. A total of 234 participants (17.9%) reported having a chronic disease diagnosis, and 218 of these individuals (93.1%) reported being diagnosed with a morbid condition that increased their risk for developing severe forms of COVID-19, based on CDC criteria [23].

The residents were from 135 institutions distributed across 25 Brazilian states. The sample consisted of medical residents (51.3%) and residents in other medical specialties (48.7%). Most participants worked at public institutions (96.7%), and 1177 residents (89.6%) worked at university hospitals. Regarding the weekly workload, 682 residents (51.9%) reported working between 60 and 90 hours. Working outside the residency programme, which was reported by 424 residents (32.3%), was more frequent among men (64.9%) than among women (57.1%). The other 889 residents (67.7%) reported not working outside the programme. Most residents (60.2%) provided direct care to patients with COVID-19 and considered the availability of PPE to be adequate (78.6%).

Regarding the degree of autonomy at work, 1089 residents (82.9%) classified their perception of autonomy as moderate to high [mean (SD) of the scores on the evaluation scale: 6.51 (2.10); 95% CI: 6.39–6.62], and 755 residents (57.5%) considered the pedagogical structure of and availability of learning resources for the residency programme moderate to good [mean (SD) of the scores: 5.77 (2.46); 95% CI: 5.64–5.91].

The DASS-21 scores for 673 (51.3%) respondents were above the cutoff for a normal state. For anxiety, 701 (53.4%) residents had high scores, and for stress, 691 (52.6%) residents had high scores. The percentage of concordant responses (indicating a frequency of occurrence of “a good part of time” or “most of the time”) was notable for the statements “I felt I wasn’t worth much as a person” (29.4% of participants) and “I felt that life was meaningless” (19.5% of participants). Based on the BRCS, 813 residents (61.9%) had low resilience. Regarding the PHQ-9, 799 residents (60.9%) had high scores. The percentage of concordant responses (indicating a frequency of occurrence of “more than half the days” or “nearly every day”) was notable for the following statements: “Little interest or pleasure in doing things?“ (43.2% of participants) and “Thoughts that you would be better off dead, or thoughts of hurting yourself in some way?” (6.8% of participants; highlighted here due to the extreme relevance of the content).

In the unadjusted bivariate analyses (supplementary material), considering the entire sample, there was a significant association between depression - DASS-21 (Table S1) and the presence of chronic diseases, perception of low autonomy, poor adequacy of the educational structure of the residency programme, inadequate PPE availability and low resilience (p < 0.001 in all cases). For the outcome anxiety - DASS-21 (Table S2), there was a significant association with the female gender (p < 0.001), working outside the residency programme (p = 0.003), presence of chronic diseases (p < 0.001), perception of low autonomy (p = 0.023), poor adequacy of the pedagogical structure (p < 0.001), inadequate PPE availability (p < 0.001) and low resilience (p < 0.001). For the outcome stress - DASS-21 (Table S3), there was a significant association with the female gender (p < 0.001), presence of chronic diseases (p = 0.001), perception of low autonomy (p < 0.001), poor adequacy of the pedagogical structure (p < 0.001), inadequate PPE availability (p < 0.001) and low resilience (p < 0.001).

Depression, as evaluated by the PHQ-9 (Table S4), was significantly associated with the female gender (p < 0.001), presence of chronic diseases (p < 0.001), perception of low autonomy (p < 0.001), poor adequacy of the pedagogical structure (p < 0.001), inadequate PPE availability (p < 0.001), workload > 60 h/week (p = 0.036) and low resilience (p < 0.001). Details of all these observed associations are available in the supplementary material. Female gender, race, cumulative weekly workload, work outside the residency programme and direct care of patients with COVID-19 were not significant in the bivariate unadjusted analyses (p ≥ 0.05) for the DASS-21 – depression; race, cumulative weekly workload and direct care of patients with COVID-19 were not significant for DASS-21 – anxiety; race, cumulative weekly workload, work outside the residency programme and direct care of patients with COVID-19 were not significant for DASS-21 – stress; and race, work outside the residency programme and direct care of patients with COVID-19 were not significant for the PHQ-9.

There was a strong internal correlation among the DASS-21 subscales and among these subscales and the PHQ-9, as follows: DASS-21 – depression vs. DASS-21 – anxiety (r = 0.64, p < 0.001); DASS-21 – depression vs. DASS-21 – stress (r = 0.72, p < 0.001); DASS-21 – anxiety vs. DASS-21 – stress (r = 0.75, p < 0.001); PHQ-9 – depression vs. DASS-21 – depression (r = 0.79, p < 0.001); PHQ-9 – depression vs. DASS-21 – anxiety (r = 0.65, p < 0.001); and PHQ-9 – depression vs. DASS-21 – stress (r = 0.73, p < 0.001).

Table 2 summarizes the results obtained from the various instruments used to evaluate the residents and compare medical and nonmedical residents.

**Table 2:** Comparisons of psychosocial characteristics and organizational structure between medical and nonmedical residents.^†^

	NONMEDICAL n (%)	MEDICAL n (%)	ODDS RATIO	95% CI		P VALUE		
				Lower	Upper			
**GENDER (1310 responses)**								
Female	549 (86.3%)	476 (70.6%)	2.625	1.983	3.474	< 0.001		
Male	87 (13.7%)	198 (29.4%)						
**RACE (1313 responses)**								
White	338 (52.9%)	440 (65.3%)	0.597	0.478	0.746	< 0.001		
Nonwhite	301 (47.1%)	234 (34.7%)						
**PRESENCE OF DISEASES (1305 responses)**								
Yes	104 (16.4%)	130 (19.4%)	0.817	0.615	1.085	0.162		
No	530 (83.6%)	541 (80.6%)						
**DASS-21 – DEPRESSION (1313 responses)**								
Abnormal	334 (52.3%)	339 (50.3%)	1.082	0.871	1.344	0.475		
Normal	305 (47.7%)	335 (49.7%)						
**DASS-21 – ANXIETY (1313 responses)**								
Abnormal	372 (58.2%)	329 (48.8%)	1.461	1.085	1.363	0.001		
Normal	267 (41.8%)	345 (51.2%)						
**DASS-21 – STRESS (1313 responses)**								
Abnormal	342 (53.5%)	349 (51.8%)	1.072	0.863	1.332	0.528		
Normal	297 (46.5%)	325 (48.2%)						
**PHQ-9 (1313 responses)**								
High	404 (63.2%)	395 (58.6%)	1.214	0.972	1.516	0.087		
Low	235 (36.8%)	279 (41.4%)						
**BRCS (1313 responses)**								
Low resilience	414 (64.8%)	399 (59.2%)	1.268	1.014	1.586	0.037		
Moderate to high	225 (35.2%)	275 (40.8%)						
**PERCEPTION OF AUTONOMY (1313 responses)**								
Moderate to high	535 (83.7%)	554 (82.2%)	1.114	0.835	1.486	0.462		
Low	104 (16.3%)	120 (17.8%)						
**ADEQUACY OF THE PEDAGOGICAL STRUCTURE (1313 responses)**								
Moderate to high	312 (48.8%)	443 (65.7%)	0.498	0.398	0.621	< 0.001		
Low	327 (51.2%)	231 (34.3%)						
**AVAILABILITY OF PPE (1313 responses)**								
Moderate to high	515 (80.6%)	517 (76.7%)	1.261	0.967	1.644	0.086		
Low	124 (19.4%)	157 (23.3%)						
**WORK OUTSIDE THE RESIDENCY PROGRAMME (1313 responses)**								
Yes	8 (1.3%)	416 (61.7%)	0.008	0.004	0.016	< 0.001		
No	631 (98.7%)	258 (38.3%)						
**DIRECT CARE OF PATIENTS WITH COVID-19 (1313 responses)**								
Yes	246 (38.5%)	544 (80.7%)	0.150	0.117	0.192	< 0.001		
No	393 (61.5%)	130 (19.3%)						
**CUMULATIVE WEEKLY WORKLOAD (1313 responses)**								
> 60 h	294 (46.0%)	478 (70.9%)	0.349	0.278	0.439	< 0.001		
≤ 60 h	345 (54.0%)	196 (29.1%)						

†Unadjusted bivariate inferential analyses using the chi-square test

BRCS: Brief Resilient Coping Scale

CI: Confidence interval

DASS-21: Depression, anxiety, and stress scale, 21 items

PHQ-9: Patient Health Questionnaire, 9 items

PPE: Personal protective equipment

There was a clear predominance of women in both types of training programs, with a higher prevalence in nonmedical residencies. There was a predominance of whites in both programmes and a higher prevalence in medical residency programmes (Table 2). The mean (SD) age of nonmedical residents was 26.44 (4.40) years and that of medical residents was 29.21 (3.36) years (p < 0.001).

There was no significant difference between medical and other health residents regarding the presence of chronic diseases, DASS-21 – depression score, DASS-21 – stress score, BRCS score (resilience) and perception of autonomy (Table 2). However, nonmedical residents had higher DASS-21-anxiety scores than medical residents, with a mean (SD) of 13.22 (10.51) vs. 10.96 (10.00), respectively, and a mean difference (MD) of 2.26 (95% CI: 1.15–3.37; p < 0.001), and a similar trend for depression, as assessed using the PHQ-9 scale, with scores of 12.31 (6.27) vs. 11.71 (6.60) and an MD of 0.60 (95% CI: -0.098–1.299; p = 0.092).

Medical and nonmedical residency programmes differed in terms of perceived PPE availability, working outside the residency programme, providing direct care to patients with COVID-19 and weekly workload (Table 2). The perception of the adequacy of the pedagogical structure was lower among nonmedical residents than among medical residents, with a mean (SD) of 5.34 (2.50) vs. 6.18 (2.36) and an MD of -0.840 (95% CI: -1.103 – -0.576; p < 0.001).

**Fig. 1:**
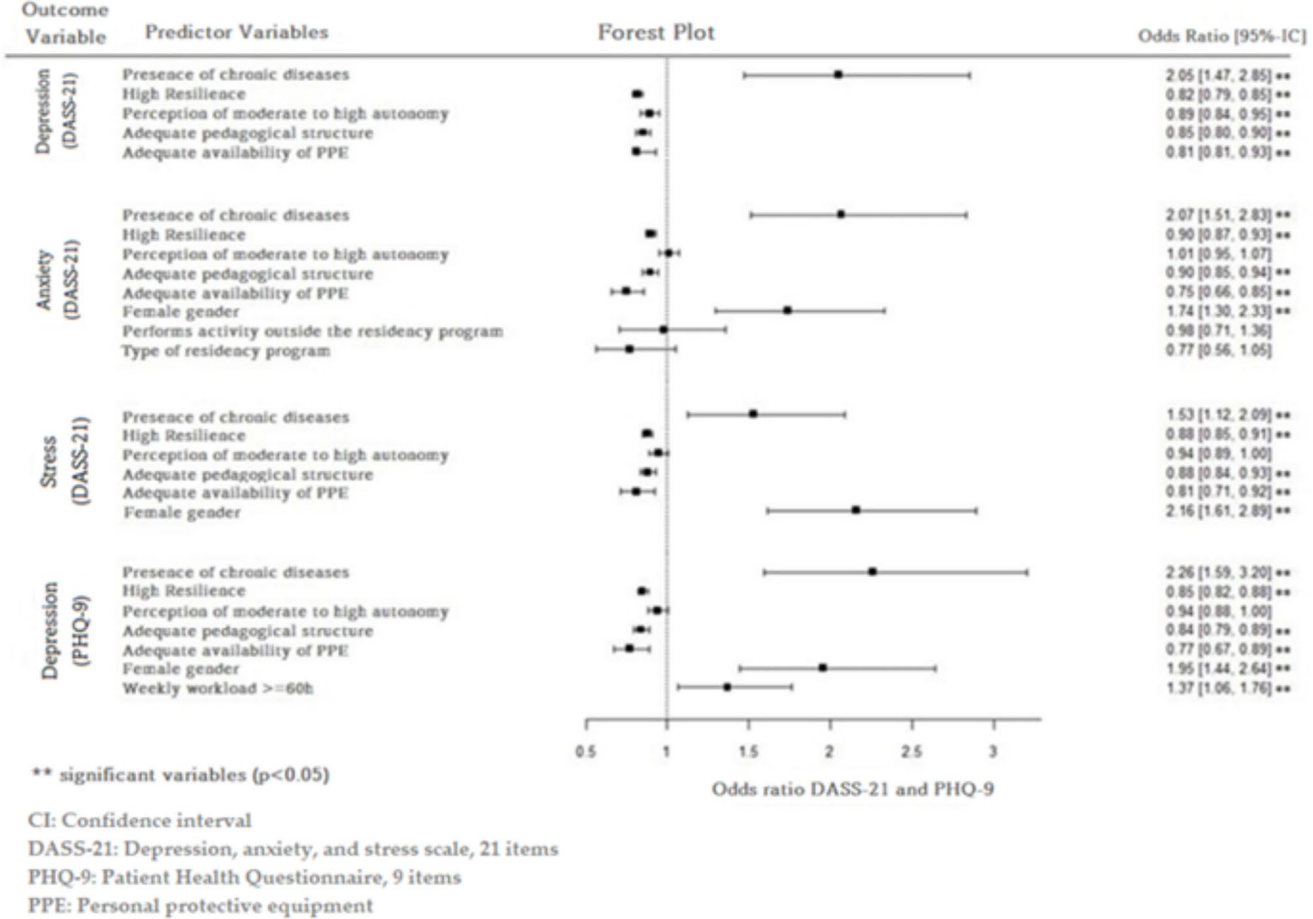
Multivariate analysis in health professionals (medical and nonmedical residents) in residency programmes during the COVID-19 pandemic **Note**: A multivariate binomial logistic regression model was fitted for each outcome variable identified in the figure

In the multivariate analysis (adjusted), the presence of chronic diseases, degree of resilience, perception of autonomy, adequacy of the pedagogical organization of the residency programme and PPE availability remained independent predictors of depression (DASS-21) (Fig. 1).

For anxiety (DASS-21), the significant independent predictor variables were the presence of chronic diseases, degree of resilience, adequacy of the pedagogical structure, availability of PPE and gender. Regarding stress (DASS-21) and depression (PHQ-9), significant independent associations were maintained for the following: presence of chronic diseases, degree of resilience, adequacy of the pedagogical structure, availability of PPE and gender. There was also a significant independent association of weekly workload with the PHQ-9 depression score (Fig. 1).

## Discussion

In the present study, the high prevalence of symptoms indicative of mental disorders among residents, found in more than 50% of residents based on DASS-21 results and in more than 60% of residents based on PHQ-9 results, stands out.

To date, few scientific studies have addressed data and intervention models focused on the mental health of health professionals in training involved in the care of patients with COVID-19 [24]. Most studies have been conducted in China, and there is a great lack of information about Latin American countries.

The present study focused on the mental health of medical and multidisciplinary residents who cared for patients diagnosed with COVID-19. A significant number of participants reported symptoms of depression, anxiety and stress, as determined using the DASS-21 scale, with 51.3% of participants reporting symptoms of depression, 53.4% reporting symptoms of anxiety and 52.6% reporting symptoms of stress. The evaluation of depressive symptoms by the PHQ-9, which is specific for this purpose, showed even higher percentages, above 60%. These values are higher than expected when considering the figures reported in other studies conducted during the pandemic involving the general population, which were approximately 15% [25]. A study conducted in the United Kingdom reported an increase in mental disorder symptoms in the general population during the pandemic (27.3%) over prepandemic periods (18.9%) [26].

The prevalence of these symptoms observed in our study was higher than that in other studies conducted with health professionals [10, 16, 27] and in the general population [28, 29] in the context of the pandemic. A multicentre study [27] with health professionals identified prevalences of 10.6%, 15.7% and 5.2% for symptoms of depression, anxiety and stress, respectively, as determined using the DASS-21 scale. Those values were similar to findings for the general population in a study conducted in Spain [28]. In studies that used the PHQ-9 for depression screening, the rates were 50.4% for health professionals [10] and 19% for the general population in Hong Kong [29]. Kannampallil et al. [30] conducted a study with physician trainees in the United States and observed prevalence rates of 28%, 22% and 29% for symptoms of depression, anxiety and stress, respectively. Other studies show that before the pandemic, depression, distress and burnout were higher among medical residents than among the general working population in the United States [31, 32].

In early 2020, a study conducted with the general population in China found that 53.8% of respondents experienced moderate to severe psychological impacts due to the consequences of the pandemic, of whom 16.5% had symptoms of depression, 28.8% had symptoms of anxiety and 8.1% had symptoms of stress [33]. High levels of anxiety and depression during the pandemic were also observed in other studies conducted in China and Spain, with rates of depression and anxiety of approximately 20–30% [28, 34]. Compared to a study involving medical residents conducted in the United States during the pandemic, with rates of depressive symptoms of approximately 21% [35], our sample maintained higher levels of the aforementioned symptoms. These data are worrisome regarding both residents’ health and the risk posed by their care activities; importantly, studies have indicated an association between depression and a greater propensity of medical errors [36].

As in other studies related to the mental health of health professionals, the majority of respondents (78.1%) were female [11, 15, 37], with an even greater predominance in nonmedical residency programmes. The average age of our participants was younger than 30 years; most self-reported as white. Studies [28, 38] have indicated a higher prevalence of symptoms of depression, anxiety and stress in individuals in the 20- to 28-year-old age group. Based on our data, we did not observe a relationship between these symptoms and age; however, our sample consisted mainly of young people, with a relatively homogeneous age distribution, making it difficult to detect differences between age groups.

The female gender in our sample was associated with a higher prevalence of symptoms of anxiety, stress and depression (DASS-21 and PHQ-9). Some mental disorders, such as depression and anxiety, are more frequent in the female population, probably due to several biological [39], cultural and social elements. Carvalho et al. [11] postulated that the burden resulting from social and family demands that expose women to double shifts predisposes females to a high prevalence of psychological distress. During the pandemic, women experienced an intensification of their daily work routines, which, among other factors, possibly contributed to the increase in mental disorder symptoms in women [37].

The presence of pre-existing chronic diseases was associated with symptoms of depression, anxiety and stress. Some studies indicate that the presence of chronic morbidities is significantly associated with higher levels of psychological symptoms, which increase in stressful situations [28, 40, 41], such as the pandemic. The scenario is even more worrisome given that in many developing countries, such as Brazil, access to mental health services is limited.

A high workload, reported by 51.9% of the study participants, is cited in studies as a predisposing factor for mental disorders. Data indicate that an extensive workload can cause discontent and suffering among residents [42], resulting in feelings of weariness, frustration and overload. To alleviate this issue, reduced workload for residents has been proposed in several countries [43, 44]; in Brazil, the official workload is 60 hours per week. Despite this limit, a substantial proportion (32.3%) of the participants work outside residency programmes, with a higher frequency reported by those in medical residency programmes (61.7%). This difference, compared to other residency programmes, is because residents of other health areas cannot work outside the residency programme [45].

We found that the better the resilience score, degree of autonomy, adequacy of pedagogical organization and availability of PPE, the lower the risk of scores indicative of depression, anxiety and stress, as determined by the DASS-21.

Resilience is strongly associated with protection against mental disorders and inversely related to the risk of developing mental disorders. Thus, even with the challenges posed by the pandemic, health professionals will experience reduced negative impacts on their mental health if they have favourable working conditions [46]. In addition, the reduced availability of resources such as PPE and the lack of information on protective measures are considered aggravating factors [47].

Based on our data, a good pedagogical structure in the residency programme (subjective evaluation by participants) was associated with a lower risk of developing mental disorder symptoms. Inadequate infrastructure and insufficient human and material resources to meet the demands of care also cause suffering among these professionals. Activities that foster the production and discussion of situations of stress and suffering are useful and little explored tools for coping with such issues in hospital settings [48].

The present study has limitations. Although there were responses from hospitals in nearly all Brazilian states, the participants were predominantly from university hospitals, which may not reflect the situation of all residents in the country. University hospitals generally share characteristics related to pedagogical organization, human resources and care infrastructure, which are eminently focused on high-complexity care. These characteristics do not necessarily apply to most nonuniversity hospitals. Thus, it will be important to expand the representativeness of residents from nonuniversity institutions and evaluate any differences in future studies.

The period of residence was not registered in our study, but all residents were almost equally involved in the care provided to patients with COVID 19. Although, it is possible that differences in previous knowledge impact on emotional stress.

The use of social networks to recruit participants may result in a selection bias because those who have a greater affinity to these means of communication respond to questionnaires more frequently. In addition, social networks themselves can act as predisposers or amplifiers of mental disorders; therefore, the preferential selection of regular users of such networks could increase the prevalence of mental disorders in the sample. However, the population of residents is typically composed of young people, among whom the use of social networks is widespread. Another possible source of selection bias is that people with anxiety, depression and stress could be more predisposed to participate in a study focusing on mental health.

Another possible selection bias is that people with anxiety, depression and stress, could be more predisposed to participate in a study focusing on mental health.

The higher number of female participants in the study may be related to a greater predisposition among women to health care in general and mental health care in particular [49]. However, the number of male participants (n = 285) was high, which supports the representativeness of the study findings for males.

The cross-sectional design of the study does not allow for establishing cause and effect relationships between the COVID-19 pandemic and the findings. However, the high prevalence of mental disorder symptoms observed in our study was higher than that reported in other studies in prepandemic periods [31, 32], suggesting, as a plausible hypothesis, that this serious public health problem has affected resident’s mental health.

To evaluate the adequacy of the pedagogical organization of the residency program and the availability of personal protective equipment (PPE) specific instruments were created to value the individual’s perception about the items. Even though the instruments used have not being validated, the data addressed had an exploratory nature and would not change the result obtained, however such instruments opened the perspective for future research as we understand that they have potential importance in the hospital academic setting.

This study was the largest ever conducted in Brazil among residents in medicine and other health specialties during the COVID-19 pandemic, and the results reveal a high prevalence of symptoms indicative of mental disorders among health professionals in training. The study had national representativeness, with a large number of participants (n = 1313) linked to 135 institutions distributed throughout nearly all Brazilian states (25 of the 27 states). The results will allow us to deepen knowledge of mental health problems in this specific population and thus contribute to planning actions to support these professionals in training.

## Conclusions

We found a high prevalence of symptoms of depression, anxiety and stress among medical and nonmedical residents during the COVID-19 pandemic in Brazil. There was an association between these symptoms and the female gender, the presence of diseases and a high weekly workload. Mental disorders are complex and have biological, social and psychological factors, and the particular academic environment of a residency, aggravated by the need to care for patients with COVID-19, may be a potential stressor and associated with the high prevalence of these symptoms among residents. This study points to the need for greater attention to these professionals and for the implementation of actions to support and promote their mental health.

## Electronic supplementary material

Below is the link to the electronic supplementary material.

Supplementary Material 1

## Data Availability

The data reported in this survey are available at https://dataverse.harvard.edu/dataset.xhtml?persistentId=doi:10.7910/DVN/XEVL3U and were published in Harvard Dataverse (view at https://dataverse.harvard.edu/dataverse/harvard). ***Competing Interests***. The authors declare that they have no competing interests.

## List of abbreviations

BRCS: Brief Resilient Coping Scale
CI: Confidence interval
DASS-21: Depression, anxiety, and stress scale, 21 items
EBSERH: Empresa Brasileira de Serviços Hospitalares
MD: Mean Difference
PHQ-9: Patient Health Questionnaire, 9 items
PPE: Personal protective equipment
SD: Standard Deviation
