# Supplementary material for: High prevalence of mental disorder symptoms among medical and other health specialties residents during the COVID-19 pandemic
Source: BMC Med Educ. 2023 May 22;23:361. doi: 10.1186/s12909-023-04202-7 (PMC10202055; doi:10.1186/s12909-023-04202-7)
Supplement: Supplementary file 1 — Supplementary Material 1 [file 12909_2023_4202_MOESM1_ESM.docx]

Supplementary Material - Bivariate Analyses

Table S1. DASS 21 - Depression. ^†^

|  | **NORMAL**  **(%)** | **ABNORMAL (%)** | **ODDS RATIO** | **95% CI** | | **CRAMER’S** **V** | **P VALUE** |
| --- | --- | --- | --- | --- | --- | --- | --- |
|  |  |  |  | **Lower** | **Upper** |  |  |
| **GENDER** |  |  |  |  |  |  |  |
| Female | 487 (47.5%) | 538 (52.5%) | 1.245 | 0.957 | 1.619 | 0.045 | 0.102 |
| Male | 151 (53.0%) | 134 (47.0%) |  |  |  |  |  |
| **RACE** |  |  |  |  |  |  |  |
| White | 393 (50.5%) | 385 (49.5%) | 1.190 | 0.955 | 1.484 | 0.043 | 0.122 |
| Non-white | 247 (46.2%) | 288 (53.8%) |  |  |  |  |  |
| **PRESENCE OF DISEASES** | | | | | | | |
| Yes | 80 (34.2%) | 154 (65.8%) | 2.078 | 1.547 | 2.793 | 0.136 | 0.000 |
| No | 556 (51.9%) | 515 (48.1%) |  |  |  |  |  |
| **PERCEPTION OF AUTONOMY** | | | | | | | |
| Moderate to high | 576 (52.9%) | 513 (47.1%) | 0.356 | 0.260 | 0.487 | 0.183 | 0.000 |
| Low | 64 (28.6%) | 160 (71.4%) |  |  |  |  |  |
| **ADEQUACY OF THE PEDAGOGICAL STRUCTURE** | | | | | | | |
| Moderate to high | 448 (59.3%) | 307 (40.7%) | 0.359 | 0.286 | 0.451 | 0.247 | 0.000 |
| Low | 192 (34.4%) | 366 (65.6%) |  |  |  |  |  |
| **AVAILABILITY OF PPE** | | | | | | | |
| Moderate to high | 545 (52.8%) | 487 (47.2%) | 0.456 | 0.346 | 0.601 | 0.156 | 0.000 |
| Low | 95 (33.8%) | 186 (66.2%) |  |  |  |  |  |
| **CUMULATIVE WEEKLY WORK LOAD** | | | | | | | |
| >60 h | 363 (47.0%) | 409 (53.0%) | 1.182 | 0.949 | 1.473 | 0.041 | 0.136 |
| ≤60 h | 277 (51.2%) | 264 (48.8%) |  |  |  |  |  |
| **WORK OUTSIDE** **THE RESIDENCY PROGRAM** | | | | | | | |
| Yes | 223 (52.6%) | 201 (47.4%) | 0.796 | 0.632 | 1.004 | 0.053 | 0.054 |
| No | 417 (46.9%) | 472 (53.1%) |  |  |  |  |  |
| **DIRECT CARE OF PATIENTS WITH COVID-19** | | | | | | | |
| Yes | 383 (48.5%) | 407 (51.5%) | 1.027 | 0.823 | 1.281 | 0.006 | 0.815 |
| No | 257 (49.1%) | 266 (50.9%) |  |  |  |  |  |
| **BRCS** | | | | | | | |
| Low resilience | 295 (36.3%) | 518 (63.7%) | 3.908 | 3.082 | 4.956 | 0.318 | 0.000 |
| Moderate to high | 345 (69.0%) | 155 (31.0%) |  |  |  |  |  |
| †Unadjusted bivariate inferential analyses using the chi-square test | | | | | | |  |

Table S2. DASS 21 - Anxiety. ^†^

|  | **NORMAL**  **(%)** | **ABNORMAL (%)** | **ODDS RATIO** | **95% CI** | | **CRAMER’S** **V** | **P VALUE** |
| --- | --- | --- | --- | --- | --- | --- | --- |
|  |  |  |  | **Lower** | **Upper** |  |  |
| **GENDER** |  |  |  |  |  |  |  |
| Female | 449 (43.8%) | 576 (56.2%) | 1.690 | 1.296 | 2.202 | 0.108 | 0.000 |
| Male | 162 (56.8%) | 123 (43.2%) |  |  |  |  |  |
| **RACE** |  |  |  |  |  |  |  |
| White | 372 (47.8%) | 406 (52.2%) | 1.126 | 0.903 | 1.405 | 0.029 | 0.292 |
| Non-white | 240 (44.9%) | 295 (55.1%) |  |  |  |  |  |
| **PRESENCE OF DISEASES** | | | | | | | |
| Yes | 75 (32.1%) | 159 (67.9%) | 2.108 | 1.562 | 2.844 | 0.137 | 0.000 |
| No | 534 (49.9%) | 537 (50.1%) |  |  |  |  |  |
| **PERCEPTION OF AUTONOMY** | | | | | | | |
| Moderate to high | 523 (48.0%) | 566 (52.0%) | 0.713 | 0.532 | 0.956 | 0.063 | 0.023 |
| Low | 89 (39.7%) | 135 (60.3%) |  |  |  |  |  |
| **ADEQUACY OF THE PEDAGOGICAL STRUCTURE** | | | | | | | |
| Moderate to high | 412 (54.6%) | 343 (45.4%) | 0.465 | 0.372 | 0.582 | 0.186 | 0.000 |
| Low | 200 (35.8%) | 358 (64.2%) |  |  |  |  |  |
| **AVAILABILITY OF PPE** | | | | | | | |
| Moderate to high | 519 (50.3%) | 513 (49.7%) | 0.489 | 0.371 | 0.645 | 0.141 | 0.000 |
| Low | 93 (33.1%) | 188 (66.9%) |  |  |  |  |  |
| **CUMULATIVE WEEKLY WORK LOAD** | | | | | | | |
| >60 h | 358 (46.4%) | 414 (53.6%) | 1.023 | 0.821 | 1.276 | 0.006 | 0.837 |
| ≤60 h | 254 (47.0%) | 287 (53.0%) |  |  |  |  |  |
| **WORK OUTSIDE** **THE RESIDENCY PROGRAM** | | | | | | | |
| Yes | 223 (52.6%) | 201 (47.4%) | 0.701 | 0.556 | 0.884 | 0.083 | 0.003 |
| No | 389 (43.8%) | 500 (56.2%) |  |  |  |  |  |
| **DIRECT CARE OF PATIENTS WITH COVID-19** | | | | | | | |
| Yes | 362 (45.8%) | 428 (54.2%) | 1.083 | 0.868 | 1.351 | 0.019 | 0.482 |
| No | 250 (47.8%) | 273 (52.2%) |  |  |  |  |  |
| **BRCS** | | | | | | | |
| Low resilience | 315 (38.7%) | 498 (61.3%) | 2.313 | 1.842 | 2.904 | 0.201 | 0.000 |
| Moderate to high | 297 (59.4%) | 203 (40.6%) |  |  |  |  |  |
| †Unadjusted bivariate inferential analyses using the chi-square test | | | | | | |  |

Table S3. DASS 21 - Stress. ^†^

|  | **NORMAL**  **(%)** | **ABNORMAL (%)** | **ODDS RATIO** | **95% CI** | | **CRAMER’S** **V** | **P VALUE** |
| --- | --- | --- | --- | --- | --- | --- | --- |
|  |  |  |  | **Lower** | **Upper** |  |  |
| **GENDER** |  |  |  |  |  |  |  |
| Female | 451 (44.0%) | 574 (56.0%) | 1.881 | 1.441 | 2.457 | 0.129 | 0.000 |
| Male | 170 (59.6%) | 115 (40.4%) |  |  |  |  |  |
| **RACE** |  |  |  |  |  |  |  |
| White | 368 (47.3%) | 410 (52.7%) | 0.993 | 0.797 | 1.238 | 0.002 | 0.950 |
| Non-white | 254 (47.5%) | 281 (52.5%) |  |  |  |  |  |
| **PRESENCE OF DISEASES** | | | | | | | |
| Yes | 88 (37.6%) | 146 (62.4%) | 1.625 | 1.216 | 2.173 | 0.091 | 0.001 |
| No | 530 (49.5%) | 541 (50.5%) |  |  |  |  |  |
| **PERCEPTION OF AUTONOMY** | | | | | | | |
| Moderate to high | 542 (49.8%) | 547 (50.2%) | 0.561 | 0.416 | 0.755 | 0.106 | 0.000 |
| Low | 80 (35.7%) | 144 (64.3%) |  |  |  |  |  |
| **ADEQUACY OF THE PEDAGOGICAL STRUCTURE** | | | | | | | |
| Moderate to high | 419 (55.5%) | 336 (44.5%) | 0.459 | 0.366 | 0.574 | 0.189 | 0.000 |
| Low | 203 (36.4%) | 355 (63.6%) |  |  |  |  |  |
| **AVAILABILITY OF PPE** | | | | | | | |
| Moderate to high | 528 (51.2%) | 504 (48.8%) | 0.480 | 0.364 | 0.632 | 0.145 | 0.000 |
| Low | 94 (33.5%) | 187 (66.5%) |  |  |  |  |  |
| **CUMULATIVE WEEKLY WORK LOAD** | | | | | | | |
| >60 h | 354 (45.9%) | 418 (54.1%) | 1.159 | 0.930 | 1.445 | 0.036 | 0.188 |
| ≤60 h | 268 (49.5%) | 273 (50.5%) |  |  |  |  |  |
| **WORK OUTSIDE** **THE RESIDENCY PROGRAM** | | | | | | | |
| Yes | 213 (50.2%) | 211 (49.8%) | 0.844 | 0.670 | 1.064 | 0.040 | 0.151 |
| No | 409 (46.0%) | 480 (54.0%) |  |  |  |  |  |
| **DIRECT CARE OF PATIENTS WITH COVID-19** | | | | | | | |
| Yes | 361 (45.7%) | 429 (54.3%) | 1.184 | 0.949 | 1.477 | 0.041 | 0.135 |
| No | 261 (49.9%) | 262 (50.1%) |  |  |  |  |  |
| **BRCS** | | | | | | | |
| Low resilience | 310 (38.1%) | 503 (61.9%) | 2.693 | 2.140 | 3.388 | 0.236 | 0.000 |
| Moderate to high | 312 (62.4%) | 188 (37.6%) |  |  |  |  |  |
| †Unadjusted bivariate inferential analyses using the chi-square test | | | | | | |  |

Table S4. PHQ-9. ^†^

|  | **LOW**  **(%)** | **HIGH**  **(%)** | **ODDS RATIO** | **95% CI** | | **CRAMER’S** **V** | **P VALUE** |
| --- | --- | --- | --- | --- | --- | --- | --- |
|  |  |  |  | **Lower** | **Upper** |  |  |
| **GENDER** |  |  |  |  |  |  |  |
| Female | 375 (36.6%) | 650 (63.4%) | 1.627 | 1.249 | 2.121 | 0.100 | 0.000 |
| Male | 138 (48.4%) | 147 (51.6%) |  |  |  |  |  |
| **RACE** |  |  |  |  |  |  |  |
| White | 301 (38.7%) | 477 (61.3%) | 0.954 | 0.761 | 1.195 | 0.011 | 0.682 |
| Non-white | 213 (39.8%) | 322 (60.2%) |  |  |  |  |  |
| **PRESENCE OF DISEASES** | | | | | | | |
| Yes | 57 (24.4%) | 177 (75.6%) | 2.302 | 1.668 | 3.178 | 0.143 | 0.000 |
| No | 456 (42.6%) | 615 (57.4%) |  |  |  |  |  |
| **PERCEPTION OF AUTONOMY** | | | | | | | |
| Moderate to high | 460 (42.2%) | 629 (57.8%) | 0.434 | 0.313 | 0.604 | 0.140 | 0.000 |
| Low | 54 (24.1%) | 170 (75.9%) |  |  |  |  |  |
| **ADEQUACY OF THE PEDAGOGICAL STRUCTURE** | | | | | | | |
| Moderate to high | 371 (49.1%) | 384 (50.9%) | 0.357 | 0.281 | 0.452 | 0.238 | 0.000 |
| Low | 143 (25.6%) | 415 (74.4%) |  |  |  |  |  |
| **AVAILABILITY OF PPE** | | | | | | | |
| Moderate to high | 441 (42.7%) | 591 (57.3%) | 0.470 | 0.351 | 0.631 | 0.141 | 0.000 |
| Low | 73 (26.0%) | 208 (74.0%) |  |  |  |  |  |
| **CUMULATIVE WEEKLY WORK LOAD** | | | | | | | |
| >60 h | 284 (36.8%) | 488 (63.2%) | 1.271 | 1.015 | 1.591 | 0.058 | 0.036 |
| ≤60 h | 230 (42.5%) | 311 (57.5%) |  |  |  |  |  |
| **WORK OUTSIDE** **THE RESIDENCY PROGRAM** | | | | | | | |
| Yes | 180 (42.5%) | 244 (57.5%) | 0.816 | 0.645 | 1.033 | 0.047 | 0.090 |
| No | 334 (37.6%) | 555 (62.4%) |  |  |  |  |  |
| **DIRECT CARE OF PATIENTS WITH COVID-19** | | | | | | | |
| Yes | 300 (38.0%) | 490 (62.0%) | 1.131 | 0.902 | 1.418 | 0.030 | 0.285 |
| No | 214 (40.9%) | 309 (59.1%) |  |  |  |  |  |
| **BRCS** | | | | | | | |
| Low resilience | 228 (28.0%) | 585 (72.0%) | 3.429 | 2.713 | 4.333 | 0.290 | 0.000 |
| Moderate to high | 286 (57.2%) | 214 (42.8%) |  |  |  |  |  |
| †Unadjusted bivariate inferential analyses using the chi-square test | | | | | | |  |
